# Morphographic Changes in the Electrocardiogram of *Colossoma macropomum* Caused by Exposure to Manganese

**DOI:** 10.3390/ijms25168910

**Published:** 2024-08-16

**Authors:** Lorena Meirelis do Nascimento, Murilo Farias dos Santos, Clarissa Araújo da Paz, Daniella Bastos de Araújo, Rayllan da Cunha Ferreira, Yris da Silva Deiga, Luana Vasconcelos de Souza, Tays Mata Câmara, Rodrigo Gonçalves dos Santos, Anara de Sousa Barbosa, Maria Klara Otake Hamoy, Anthony Lucas Gurgel do Amaral, Luciana Eiró-Quirino, Tárcio dos Santos Cabral, Maria Adrina Paixão de Souza da Silva, Nilton Akio Muto, Moisés Hamoy

**Affiliations:** 1 Laboratory of Pharmacology and Toxicology of Natural Products, Institute of Biological Sciences-ICB/UFPA, Belém 66077-830, PA, Brazilgurgelanthony@gmail.com (A.L.G.d.A.);; 2 Metallic Materials Characterization Laboratory, Belém 66075-110, PA, Brazil; 3 Materials Processing Research Group, Belém 66075-110, PA, Brazil; 4 Center for the Valorization of Bioactive Compounds from the Amazon, Institute of Biological Sciences, Federal University of Pará, Belém 66077-830, PA, Brazil

**Keywords:** manganese, ECG, *Colossoma macropomum*, morphographic changes

## Abstract

Manganese (Mn^2+^) is an abundant chemical element in the earth’s crust and is present in soil, water, and industrial environments, including mining, welding, and battery manufacturing. Manganese (Mn) is an essential metal needed as a cofactor for many enzymes to maintain proper biological functions. Excessive exposure to Mn in high doses can result in a condition known as manganism, which results in disorders of the neurological, cardiac, and pulmonary systems. The aim of this study was to assess cardiac susceptibility to manganese intoxication in *Colossoma macropomum* subjected to a fixed concentration of 4 mg/mL for a period of up to 96 h. This study used 45 Tambaquis (30.38 ± 3.5 g) divided into five groups of 9 animals/treatment. The treated groups were exposed to the manganese concentration for a period of 24, 48, 72, and 96 h, after which the animals’ ECGs were recorded, showing heart rate, R-R interval, P-Q interval, QRS complex duration and S-T interval. The results showed that cardiac activity decreased as the contact time increased, with an increase in the P-Q and S-T intervals. This indicates that the breakdown of circulatory homeostasis in these animals was caused by contact time with manganese.

## 1. Introduction

Manganese is a metal that acts as an essential cofactor for a variety of enzyme systems. A notable example of this importance lies in the activation of mitochondrial superoxide dismutase (SOD), a key enzyme in the oxidative stress pathway in cells [1]. When it is ingested directly, it is absorbed by the enterocytes in the intestine as divalent ions (Mn^2+^) [2], penetrating the cells in the form of a cation by passive diffusion or active transport, using the proton gradient to transport metals through the cell membrane [3]. This process is important since mitochondria play a critical role in providing the energy necessary for proper functioning, especially for muscle tissue [4].

Environmental exposure to manganese poses risks to human health, with elements that are still poorly understood [5]. Chronic inhalation of manganese in welding vapor has been associated with decreased neurological function [6]. Many studies have been developed to analyze the association between environmental exposure to Mn and health effects, most of them including the measurement of Mn in selected human biomarkers [7]. Manganese (Mn) can pass through the placenta, so exposed populations may be subjected to considerable levels in utero [8]. The complexity of establishing exposure limits to Mn is partly related to its dual role as an essential micronutrient, with low levels required for good health, but also as a neurotoxin at elevated levels. Therefore, the introduction of new techniques to recognize biomarkers of environmental exposure to manganese (Mn) through electrophysiological tools can clarify the effects of manganese in environments on various aquatic organisms using *Colossoma macropomum* (Tambaqui) as a model [9,10,11,12,13,14].

Manganese divalent cation (Mn^2+^) is sequestered by the mitochondria of the liver, brain, and heart via the mitochondrial Ca^2+^ transporter. The binding of Ca^2+^ to the activation site greatly increases the absorption rate of Ca^2+^ and Mn^2+^. Mn^2+^ is transported out of the mitochondria via a very slow energy-dependent mechanism. Consequently, Mn^2+^ is not significantly transported via the Na^+^-dependent efflux mechanism, which is the dominant efflux mechanism in the mitochondria of the heart and brain. This slow outflow of Mn^2+^ can influence mitochondrial function in these tissues [15].

When the energy support decreases for the cell due to injuries, dysfunctions, or diseases, the heart’s ability to generate the necessary energy is compromised, resulting in changes identified through electrocardiogram (ECG) graph elements, by monitoring the rhythm of heartbeats, heart rate, and identification of alterations in ECG elements [16,17]. The application of electrocardiography for the analysis of cardiac function in fish can be performed based on the characterization of electrocardiographic patterns and tracings, such as QT and RR wave intervals, QRS complex amplitude, in addition to heart rate [10]. Thus, it can be used as a biomarker for contaminants that can alter cardiac function, such as manganese. With this purpose, we used the cardiac electrophysiology of *Colossoma macropomum* (Tambaqui), a species endemic to the Amazon and Orinoco River basins, along with their tributaries, being a frequent representative in floodplain areas. It is of considerable economic relevance to several nations in northern Latin America. This fish exhibits remarkable adaptability to farming environments, as well as resistance to diseases, attributes that have played a fundamental role in optimizing aquaculture practices [18,19].

The aim of this study was to analyze the breakdown of cardiac homeostasis in a controlled environment contaminated with manganese at a known concentration for a period of up to 96 h of contact to evaluate electrocardiographic biomarkers to demonstrate cardiac toxicity.

## 2. Results

**Figure 1:**
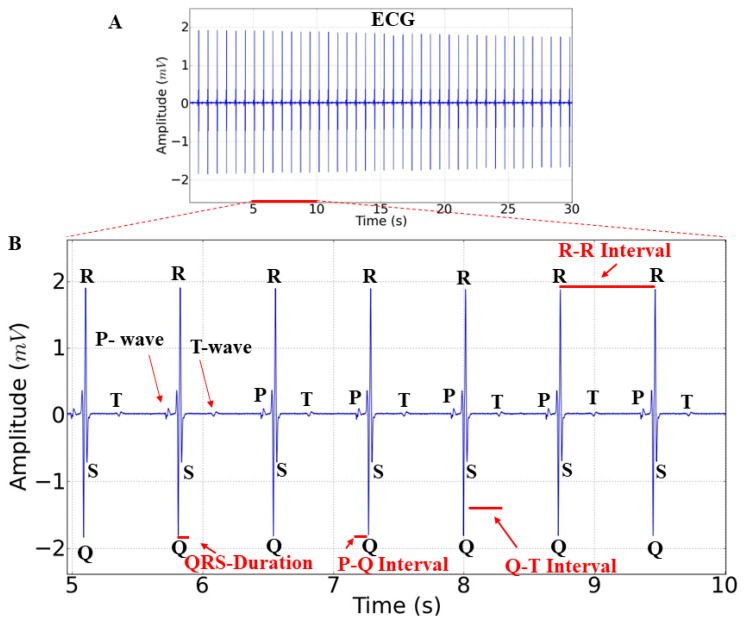
Electrocardiographic (ECG) recordings of the control group in Tambaqui, *Colossoma macropomum* (**A**). Amplification of the control group ECG with a duration of 5 s (5–10 s) showing the identification of wave elements (P wave, QRS complex, and T wave) and the analyzed parameters, as follows: heart rate, P-Q interval, R-R interval, QRS duration, and S-T interval (**B**).

The normal electrocardiogram of *Colossoma macropomum* (Figure 1A) displays a regular heart rhythm and ECG graphological elements, such as the P wave, QRS complex, and T wave (Figure 1B). The ECG characteristics analyzed during this study include the time to the onset of ventricular depolarization (Q wave) and ventricular repolarization (T wave), represented by the S-T interval. Considering the normal cardiac parameters measured during the amplified recording period (5 s) (marked in red), we identified the P-Q, R-R, and S-T intervals (Figure 1B).

**Figure 2:**
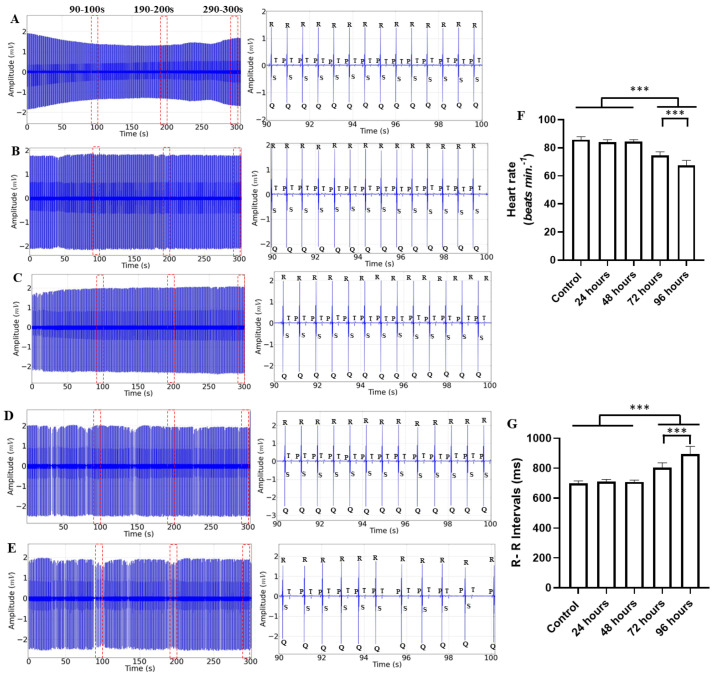
Electrocardiographic (ECG) recordings of *Colossoma macropomum* lasting 300 s and their amplifications (90–100 s) during contact with 4 mg/L of manganese acetate for the following groups: control (**A**); group treated for 24 h (**B**); group treated for 48 h (**C**); group treated for 72 h (**D**); group treated for 96 h (**E**); average heart rate (bpm) during each treatment during the 90–100 s interval (**F**); and average R-R interval (ms) for the treatments, during the 90–100 s interval (**G**). [ANOVA followed by Tukey’s test (* *p* < 0.05 ** *p* < 0.01 and *** *p* < 0.001 n = 9)].

The ECG measurements of animals treated with various manganese contact times manifested cardiac signs starting at 72 h, with a decrease in heart rate and a consequent increase in RR interval. In all records, 10 s interval periods were selected and marked in red dotted lines (Figure 2, left). The morphographic elements can be identified with little variation in the amplification of the recordings (Figure 2 Center) in Figure 2A–C, but the rhythm is modified in the recordings in which the treatment was 72 h and 96 h (Figure 2D,E). The mean heart rates were similar for the control group (85.78 ± 2.10 bpm), the 24 h treatment group (84.22 ± 1.56 bpm) (*p* = 0.644), and the 48 h treatment group (84.44 ± 1.33 bpm) (*p* = 0.761). The groups of animals treated for 72 h (74.67 ± 2.44 bpm) and 96 h (67.33 ± 3.74 bpm) showed a decrease in heart rate, with more intense changes in the group treated for 96 h (Figure 2F). The mean RR interval for the control group (699.3 ± 17.04 ms), the group treated for 24 h (712.1 ± 13.29 ms) (*p* = 0.882), and the group treated for 48 h (709.9 ± 11.25 ms) (*p* = 0.937) were similar. The groups treated for 72 h (805.4 ± 30.64 ms) and treated for 96 h (893.4 ± 51.85 ms) were greater than the other groups (Figure 2G).

**Figure 3:**
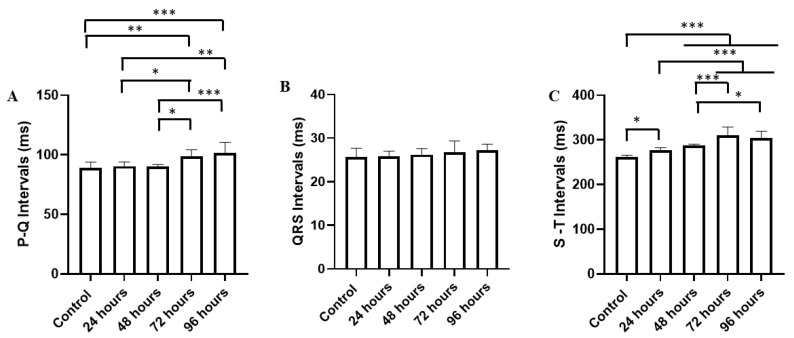
Recordings showing cardiac activity over 10 s (90–100 s) for the groups treated with manganese at different exposure times: mean P-Q interval (ms) (**A**); mean QRS complex duration (ms) (**B**); mean S-T interval (ms) (**C**). [ANOVA followed by Tukey’s test (* *p* < 0.05 ** *p* < 0.01 and *** *p* < 0.001 n = 9)].

The ECG graphic components in the period of 90–100 s relative to the average PQ interval for the control group (89.11 ± 4.70 ms) were similar to the groups treated for 24 h (90.44 ± 3.46 ms) (*p* = 0.985) and treated for 48 h (90.11 ± 1.69 ms) (*p* = 0.995). The groups treated for 72 h (98.67 ± 5.47 ms) and treated for 96 h (101.3 ± 9.16 ms) were higher than the other groups (Figure 3A).

The average QRS complex duration during the control was (25.67 ± 2.00 ms). When compared to the other groups treated for 24 h (25.89 ± 1.10 ms), 48 h (26.22 ± 1.39 ms), 72 h (26.78 ± 2.58 ms), and 96 h (27.22 ± 1.39 ms), all groups were similar (F(4, 40) = 1.157; *p* = 0.344) (Figure 3B).

The average S-T interval for the control group (262.0 ± 3.74 ms) was lower than the other groups treated for 24 h (277.3 ± 5.19 ms), 48 h (288.2 ± 2.43 ms), 72 h (310.9 ± 18.00 ms), and 96 h (303.7 ± 15.44 ms). The groups treated for 72 and 96 h showed a longer S-T interval (Figure 3C).

**Figure 4:**
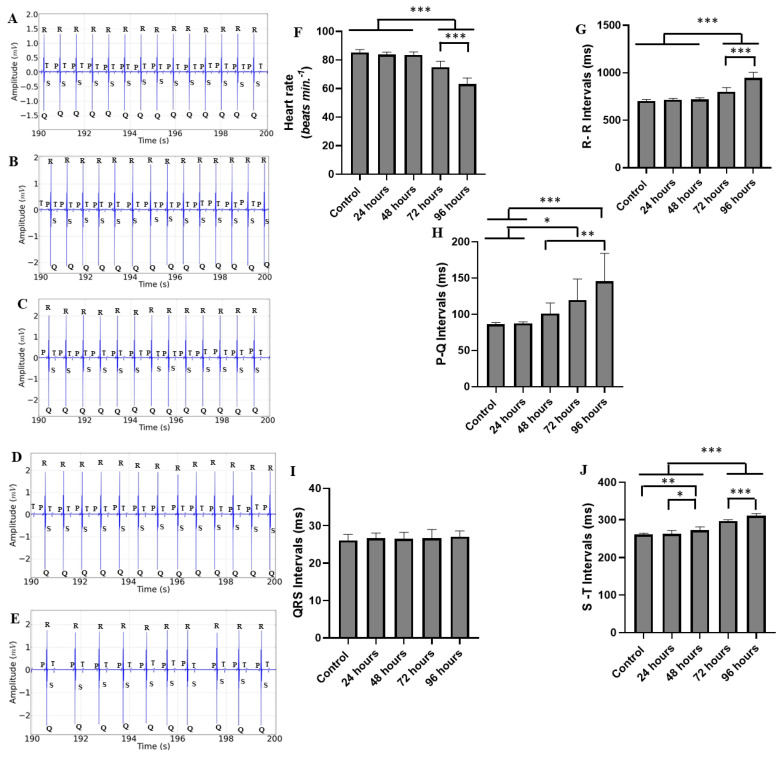
Recordings showing cardiac activity in the interval (190–200 s) of groups with different exposure times to manganese: control group (**A**); group treated for 24 h (**B**); group treated for 48 h (**C**); group treated for 72 h (**D**); group treated for 96 h (**E**); heart rate averages (bpm) (**F**); R-R interval averages (ms) (**G**); P-Q interval averages (ms) (**H**); QRS complex duration average (ms) (**I**); and S-T interval averages (ms) (**J**). [ANOVA followed by Tukey’s test (* *p* < 0.05 ** *p* < 0.01 and *** *p* < 0.001 n = 9)].

The heart of the Tambaqui during the recording period of 190 to 200 s showed little variation compared to the first period; however, there were changes not observed in the first period. The trace segments demonstrated a decrease in cardiac activity, with pronounced arrhythmia for the group treated for 72 h (Figure 4A–E). The average heart rates were similar for the following groups: control (85.78 ± 2.10 bpm), 24 h treatment group (83.78 ± 1.85 bpm) (*p* = 0.820), and 48 h treatment group (83.56 ± 2.18 bpm) (*p* = 0.735). The groups of animals treated for 72 h (75.11 ± 4.014 bpm) and 96 h (63.11 ± 43.72 bpm) showed a decrease in heart rate, maintaining a pattern similar to the first period (Figure 4F).

The average RR interval for the control group (703.0 ± 16.13 ms) was similar to the groups treated for 24 h (716.1 ± 16.29 ms) (*p* = 0.938) and 48 h (718.1 ± 16.29 ms) (*p* = 0.904). For the groups treated for 72 h (800.7 ± 43.32 ms) and 96 h (944.0 ± 61.91 ms), the intervals were longer than the other groups, with a significant increase in the 96 h group attributed to the worsening arrhythmia (Figure 4G).

The ECG graphic components, in relation to the average PQ interval during the period of 190–200 s for the control group (86.22 ± 2.53 ms), were similar to the groups treated for 24 h (87.33 ± 2.23 ms) (*p* = 0.999) and 48 h (101.1 ± 14.55 ms) (*p* = 0.631). The groups treated for 72 h (119.8 ± 28.87 ms) and 96 h (145.4 ± 38.62 ms) had longer intervals than the other groups. However, the group treated for 48 h was similar to the group treated for 72 h (*p* = 0.413) (Figure 4H).

The average QRS complex duration for the control group was (26.11 ± 1.61 ms). When compared to the other groups, the durations were similar, as follows: 24 h treatment group (26.67 ± 1.41 ms), 48 h treatment group (26.56 ± 1.74 ms), 72 h treatment group (26.67 ± 2.39 ms), and 96 h treatment group (27.11 ± 1.53 ms). All groups were similar (F(4, 40) = 0.363; *p* = 0.833) (Figure 4I).

The average S-T interval for the control group (261.2 ± 3.63 ms) was shorter than the groups treated for 24 h (263.3 ± 8.88 ms), 48 h (273.6 ± 7.60 ms), 72 h (296.8 ± 3.80 ms), and 96 h (310.8 ± 6.09 ms). It was observed that longer treatment durations resulted in an increased S-T interval (Figure 4J).

**Figure 5:**
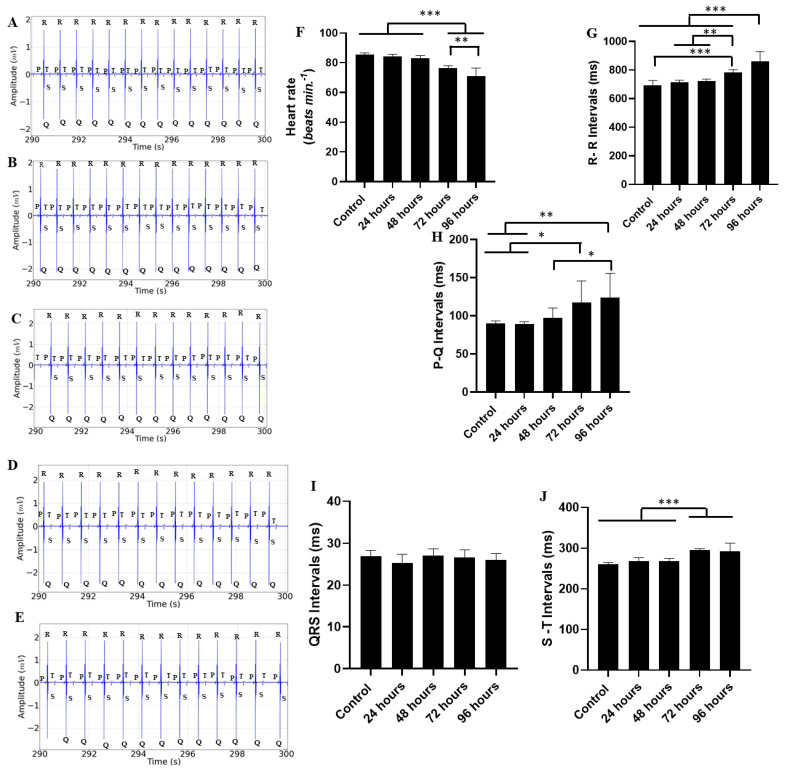
Recordings showing cardiac activity in the interval (290–300 s) during different times of exposure to manganese, as follows: control group (**A**), group treated for 24 h (**B**); group treated for 48 h (**C**); group treated for 72 h (**D**); group treated for 96 h (**E**); heart rate averages (bpm) (**F**); R-R interval averages (ms) (**G**); P-Q interval averages (ms) (**H**); QRS complex duration average (ms) (**I**); and S-T interval averages (ms) (**J**). [ANOVA followed by Tukey’s test (* *p* < 0.05 ** *p* < 0.01 and *** *p* < 0.001 n = 9)].

The cardiac activity of the Tambaqui during the recording period of 290 to 300 s showed changes similar to the previous periods. The trace segments for the groups demonstrated a decrease in cardiac activity, with an increase in arrhythmia for the groups treated for 72 and 96 h (Figure 5A–E). The average heart rates were similar for the groups, as follows: control (85.33 ± 1.41 bpm), 24 h treatment group (84.00 ± 1.73 bpm) (*p* = 0.864), and 48 h treatment group (83.11 ± 1.76 bpm) (*p* = 0.489). The groups treated for 72 h (76.44 ± 1.66 bpm) and 96 h (70.89 ± 5.57 bpm) showed a decrease in heart rate (Figure 5F).

The average RR interval for the control group (692.8 ± 34.19 ms) was similar to the groups treated for 24 h (714.0 ± 14.72 ms) (*p* = 0.716) and 48 h (721.8 ± 15.44 ms) (*p* = 0.431). However, for the groups treated for 72 h (784.8 ± 17.04 ms) and 96 h (861.7 ± 66.78 ms), the intervals were longer than the other groups, especially in the group treated for 96 h (Figure 5G).

The ECG of the control group showed an average PQ interval during the period of 190–200 s of 90.00 ± 3.64 ms, which was similar to the groups treated for 24 h (89.33 ± 2.95 ms) (*p* = 0.999) and 48 h (97.22 ± 13.23 ms) (*p* = 0.939). The groups treated for 72 h (117.4 ± 28.54 ms) and 96 h (124.3 ± 31.69 ms) had longer intervals than the other groups (Figure 5H). However, the group treated for 48 h was similar to the group treated for 72 h (*p* = 0.225).

The QRS complex for the control group (26.89 ± 1.45 ms) was similar to the groups treated for 24 h (25.33 ± 2.06 ms), 48 h (27.0 ± 1.73 ms), 72 h (26.56 ± 1.87 ms), and 96 h (26.00 ± 1.58 ms). All groups were similar F (4, 40) = 1.397; *p* = 0.252) (Figure 5I).

The average S-T interval for the control group (260.7 ± 4.21 ms) was similar to the groups treated for 24 h (267.7 ± 9.08 ms) (*p* = 0.645) and 48 h (267.7 ± 7.38 ms) (*p* = 0.645). For the groups treated for 72 h (295.2 ± 3.83 ms) and 96 h (292.1 ± 20.30 ms), it was observed that longer treatment durations increased the S-T interval in all animals, with the greatest variation in the group treated for 96 h (Figure 5J).

## 3. Discussion

Understanding the effects of manganese toxicity on aquatic animals is necessary due to factors such as human activity in mining, which can increase the number of contaminants like Mn^2+^ in the aquatic ecosystem, considering their ability to accumulate in tissues such as gills, muscles, and skin [20,21]. How this impact can be caused is not fully understood; however, there are indications that the site of intoxication would be in the plasma membranes of myocytes and mitochondria due to oxidative damage observed in these organelles [22,23,24,25,26,27]. Studies using different fish species, such as *Astyanax lacustris* and *Colossoma macropomum*, at varying concentrations are employed to understand manganese intoxication in animals [28,29].

Therefore, mitochondrial intoxication by manganese can cause systemic effects, as identified in the present study by the time-dependent impact on cardiac functionality. Mn toxicity through occupational and dietary overexposure primarily affects the central nervous system, although pulmonary, cardiac, hepatic, reproductive, and fetal toxicities have also been observed. Mn neurotoxicity results from the accumulation of the metal in brain tissue, leading to a progressive extrapyramidal disorder similar to Parkinson’s disease, with changes in the autonomic nervous system that can trigger reflex actions in the heart [30,31,32,33]. Considering the fixed concentration of 4 mg/mL of the compound over 96 h, there was a gradual increase in manganese toxicity over the marked periods, as evidenced by increasingly pronounced electrocardiographic changes in the records of each corresponding group. It is important to note that arrhythmias were identified in ECG segments with more frequent manifestations the longer the contact time with manganese.

Thus, there was a decrease in heart rate over the delimited time, revealing a progressive process of cardiac intoxication caused by manganese, confirming its cardiac toxicity in aquatic animals by interfering with cardiac homeostasis. According to Lamonzie [34] and Bers [35], at micromolar concentrations, manganese can block L-type calcium channels, leading to a decrease in action potential and calcium transition amplitude in cardiac cells, which reflects poor cardiac function. Such a situation at doses of 4 mg/L can cause a systemic effect, resulting in decreased heart rate as identified in the present results. Additionally, in vitro studies have identified that the reduction in heart contraction can occur due to manganese accumulation in tissues, while Mg^2+^ and Ca^2+^ contents decrease, promoting a negative chronotropic effect [36].

Despite the maintenance of sinus rhythm over 96 h of exposure, it was possible to analyze the alteration of the P-Q interval, which progressively increased over the study period. This was particularly evident in the recordings from the 190–200 s intervals, in which the increase in the P-Q interval after 96 h compared to all other groups indicates manganese interference in the conduction of the stimulus from the sinoatrial node to the atrioventricular node. The conduction velocity of the ventricles, represented by the duration of the QRS complex, showed no change in ventricular conduction through the bundle of His and Purkinje fibers. However, there was an increase in the S-T interval, indicating a longer time for cardiac repolarization. Although our study did not find a significant alteration in ventricular conduction, other studies suggest a cardiotoxic effect with ventricular impairment, indicating a delay in the action potential of cardiac myocytes caused by manganese accumulation in tissues [24,37,38].

Therefore, it can be concluded that manganese exposure for 72 h or more at a fixed concentration caused alterations in the heart rhythm of *Colossoma macropomum*. This suggests that the disruption of circulatory homeostasis in these animals is related to the duration of manganese exposure. Further studies are necessary to better understand the environmental impact of manganese, particularly in mining areas where river fauna may be at risk of contamination.

## 4. Material and Methods

### 4.1. Study Animals

We used 45 (n = 45) of the Tambaqui species *Colossoma macropomum*, acquired from the Tropical Species Aquaculture Laboratory (IFPA—Castanhal). The animals were stocked in aquariums in the Experimental Bioterium of the Laboratory of Pharmacology and Toxicology of Natural Products at the Federal University of Pará (UFPA) in a controlled temperature environment (25 to 27 °C) and a photoperiod of 12 h C: 12 h E. They were fed twice a day with formulated fish feed (32% protein) until satiety. The water was partially renewed (approximately 20% of the volume of the tanks) with water of the same origin while simultaneously siphoning it to remove uneaten food and feces. During acclimatization (15 days), water quality variables such as water temperature (°C), hydrogen potential (pH), dissolved oxygen (DO), ammonia (NH^3+^), and total hardness were monitored (CEUA-UFPA) 6414241023 (ID 002402).

In our research, the use of animals was carried out with the utmost respect for the ethical principles of the three Rs, as follows: replace, reduce, and improve. We ensured that animal testing was minimized whenever possible by using alternative methods. Every effort has therefore been made to reduce the number of animals used to the minimum necessary to obtain reliable results. In addition, perfecting our procedures to minimize animal discomfort and distress was a top priority throughout this study. By adhering to these principles, we strive to maintain ethical standards while advancing scientific knowledge in a responsible manner.

### 4.2. Experimental Design

#### 4.2.1. Manganese Experiment

Juvenile Tambaqui (30.38 ± 3.5 g) were randomly assigned to the following treatments: (a) control; (b) group submitted to immersion in Mn 4 mg/mL for 24 h; (c) group submitted to immersion in Mn 4 mg/mL for 48 h; (d) group submitted to immersion in Mn 4 mg/mL for 72 h; and (e) fish submitted to immersion in Mn 4 mg/mL for 96 h. All recordings lasted 5 min. For each recording, n = 9/treatment were used.

In this manuscript, the dose used was the comfortable dose for this species that was found after pilot tests with various doses, which were doses in the literature that showed that manganese causes adverse effects.

#### 4.2.2. Electrocardiogram Analysis

For cardiac monitoring, electrodes were constructed from stainless steel in a non-conjugated manner, with a diameter of 0.3 mm and a length of 5.0 mm. The positioning of the reference electrode followed the indication of the cardiac vector, being fixed on the ventral part of the left operculum (0.2 mm before the termination of the opercular cavity), while the recording electrode was inserted on the ventral part of the right operculum (2.0 mm below the pectoral fin). Subsequently, the electrodes were connected to a high-impedance amplifier. From the recordings, the following parameters were analyzed: heart rate (bpm), QRS duration (s), P-Q interval, R-R interval (s), and Q-T interval (s).

#### 4.2.3. Recording and Analysis

The electrodes were connected to a digital data acquisition system via a high-impedance input differential amplifier (Grass Technologies, Model P511, Austin, TX, USA) configured with a bandpass filtering set at 0.3 and 300 Hz and amplification at 2000× and monitored using an oscilloscope (ProteK, Model 6510, Seattle, WA, USA). The recordings were continuously digitized at a rate of 1 KHz on a computer equipped with a data acquisition board (National Instruments, Austin, TX, USA) and stored on a hard drive for subsequent processing using specialized software (LabVIEW Express, version 2011, part number 79448-35, serial number M77X/807).

The acquired signals were analyzed using a tool built in the Python programming language, version 2.7. The Numpy and Scipy libraries were used for mathematical processing, and the Matplolib library was used to produce the graphs. The graphical interface was developed using the PyQt4 library [12]. Amplitude graphs depict potential disparities between reference and recording electrodes. Signal observation from the recordings occurred at a rate of 1000 samples per second.

#### 4.2.4. Statistical Analysis

After verifying adherence to the assumptions of normality and homogeneity of variances through the Kolmogorov–Smirnov and Levene tests, respectively, the mean power values were compared using one-way ANOVA, followed by Tukey’s test. GraphPad Prism^®^ 8 software will be employed for the analyses, with significance levels set at * *p* < 0.05, ** *p* < 0.01, and *** *p* < 0.001 for all cases.

## 5. Conclusions

Based on the results obtained in this study, exposure to manganese in *Colossoma macropomum* caused significant changes in cardiac activity, as observed in changes in the P-Q and S-T intervals, as well as a reduction in heart rate over the time of exposure. These changes indicate a dysfunction in the fish’s circulatory homeostasis, exacerbated by prolonged contact with manganese. The data obtained suggest that manganese, in high concentrations and over prolonged periods, can be toxic to the cardiac system of aquatic organisms. This study contributes to understanding the impacts of manganese contamination on the aquatic environment, highlighting the importance of monitoring and regulating the levels of this metal in natural and industrial habitats to protect the health of aquatic species. In addition, the results obtained reinforce the use of electrocardiographic parameters as effective biomarkers for assessing the environmental toxicity of metals.

## Data Availability

We would like to inform you that the raw data related to our study will be available for consultation upon plausible request. We understand the importance of transparency and the possibility of replicating the results obtained, as well as the value that such data may have for future research and analysis. Upon receiving the request, our team will review it to ensure that it meets the established criteria and contact you to follow up on the request. If approved, the data will be sent in an appropriate format, along with any necessary documentation for its correct interpretation.
